# Digital intelligence technology: new quality productivity for precision traditional Chinese medicine

**DOI:** 10.3389/fphar.2025.1526187

**Published:** 2025-04-08

**Authors:** Junqing Zhu, Xiaonan Liu, Peng Gao

**Affiliations:** Shandong Key Laboratory of Digital Traditional Chinese Medicine, Institute of Pharmaceutical Research, Shandong University of Traditional Chinese Medicine, Jinan, China

**Keywords:** traditional chinese medicine, artificial intelligence, quality assessment, disease treatment, precision medicine

## Abstract

Traditional Chinese medicine is a complex medical system characterized by multiple metabolites, targets, and pathways, known for its low drug resistance and significant efficacy. However, challenges persist within Traditional Chinese medicine, including difficulties in assessing the quality of Botanical drugs, reliance on experiential knowledge for disease diagnosis and treatment, and a lack of clarity regarding the pharmacological mechanisms of Traditional Chinese medicine. The advancement of digital intelligence technology is driving a shift towards precision medicine within the Traditional Chinese medicine model. This transition propels Traditional Chinese medicine into an era of precision, intelligence, and digitalization. This paper introduces standard digital intelligence technologies and explores the application of digital intelligence technologies in quality control and evaluation of Traditional Chinese medicine, studies the research status of digital intelligence technologies in assisting diagnosis, treatment and prevention of diseases, and further promotes the application and development of digital intelligence technologies in the field of Traditional Chinese medicine.

## 1 Introduction

Traditional Chinese medicine (TCM) represents the extensive knowledge and experience gained from centuries of clinical practice. It constitutes a comprehensive medical system rooted in the principles of yin-yang, the five elements, and the interconnections between the body’s meridians, channels, and internal organs. TCM plays a vital and indispensable role in safeguarding the health of the Chinese people (Chu et al., 2020). According to TCM theory, the world is viewed as a unified entity composed of various elements. The concept of yin and yang, along with the five elements, is believed to be inherent in the human body. It is posited that disease arises from an imbalance of yin and yang within the body. The TCM theory focuses on maintaining the human body’s balance of yin and yang to address illnesses effectively. As the research model of modern medicine gradually shifts from “reductionism” to “holism”, the research strategy for diagnosing and treating diseases gradually shifts from a focus on individual diseases, targets, and medications to a more comprehensive strategy centred on system regulation and multi-target interventions (Wang et al., 2022a). TCM focuses on personalized diagnosis and treatment by targeting multiple metabolites, pathways, and factors to address low drug resistance. It aims to balance the body’s yin and yang to enhance immunity based on specific disease causes, mechanisms, and individual variations (Chen et al., 2023). Through the customization of treatment regimens, the objective is to prevent diseases and mitigate the ageing process effectively. In 2019, the global spread of the highly contagious novel coronavirus led to numerous infections and fatalities. TCM experts promptly identified and developed a set of effective treatments known as the “three medicines and three prescriptions.” These treatments, including Golden Flower Clear Sense Pellet, Lianhua Clear Plague Capsule, Haematopoietin Injection, Lung Clearing and Detoxification Soup, Dampness Corrupting Formula, and Pneumonia Corrupting Formula, demonstrated significant efficacy in combating the virus. This approach emerged as a leading treatment method in China’s epidemiological efforts, showcasing remarkable therapeutic outcomes and serving as a prominent feature in the country’s epidemic prevention and control strategies (Chen and Weidong, 2022; Ruchawapol et al., 2022). It has become one of the highlights in preventing and controlling the epidemic in China.

The challenges encountered in TCM primarily revolve around two aspects: First, medical practitioners commonly utilize the method of “identification of symptoms and quality” to evaluate the efficacy of Chinese botanical drugs. However, with substantial individual variations, quantifying these assessments may not be suitable. Modern testing technology relies on a single metabolite to determine the quality, which may not provide a comprehensive evaluation of the quality of Botancial drugs (Li X et al., 2022). Secondly, the diagnosis and treatment of diseases in TCM are influenced by TCM experience. The four diagnostic methods (observation, olfaction, inquiry, and palpation) rely on the subjective experience of TCM practitioners (Guo et al., 2020). The uncertainty of TCM language, the vagueness of TCM theories and the non-interpretability of TCM constrains the development of TCM. With the continuous implementation of China’s information technology and intelligence strategy, the application of modern technology in medicine promotes the gradual transformation of TCM into precision medicine. Precision TCM utilizes personalized medical care by incorporating genomics, proteomics, and thorough data analysis to achieve tailored and accurate forecasting, prevention, diagnosis, and treatment of illnesses. The use of digital intelligence technology is increasingly recognized as a valuable support tool in precision medicine. It aids TCM practitioners in diagnosing illnesses through observation, olfaction, inquiry, and pulse-taking, leading to cost savings and enhancing the effectiveness and safety of medical diagnosis and treatment (Gao, 2023). The technology has become an essential tool to assist TCM practitioners in judging conditions, saving medical costs, and improving diagnosis and treatment efficiency.

**FIGURE 1:**
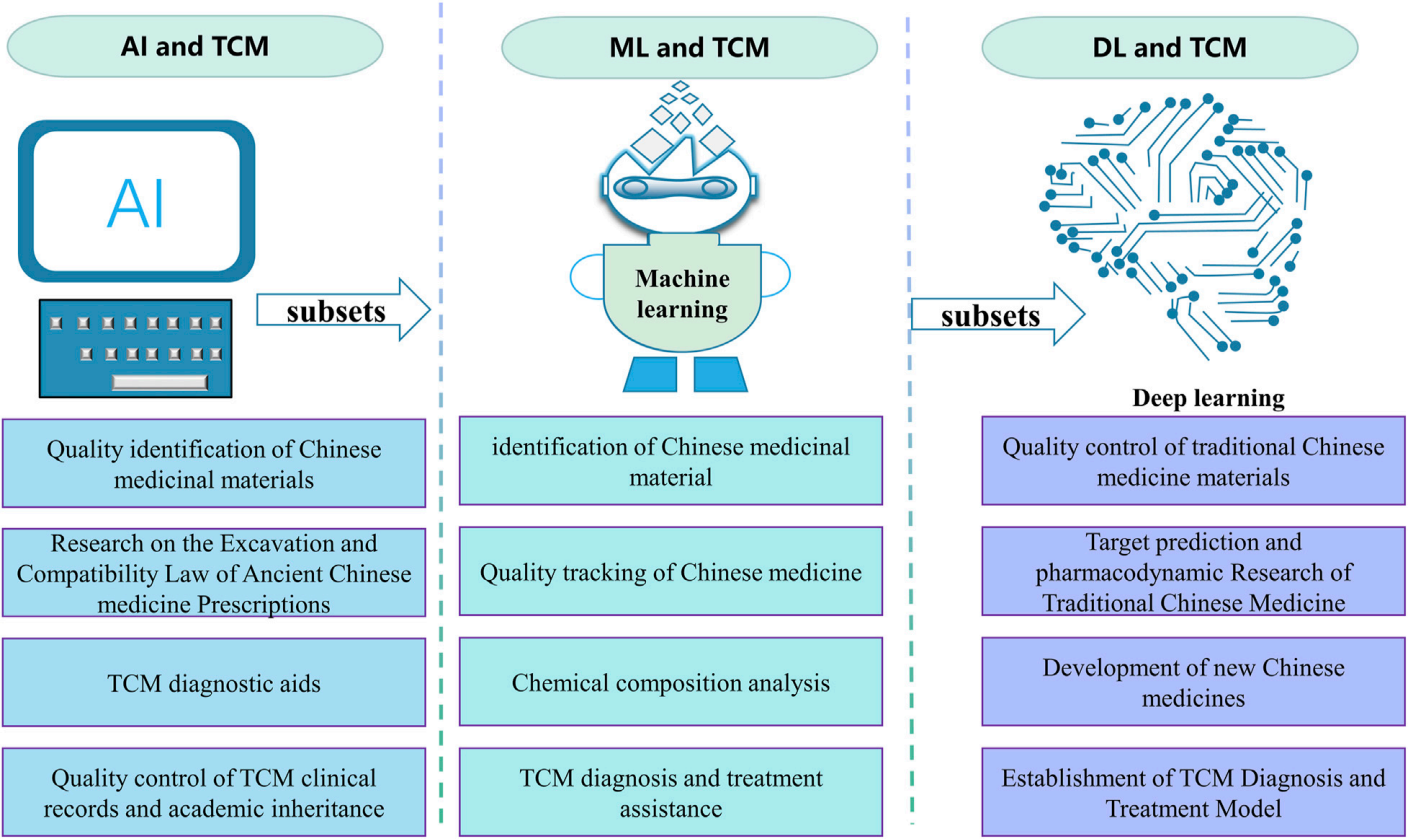
Numerical intelligence technology: A new qualitative productivity for TCM to treat diseases.

This paper reviews the standard digital intelligence technologies, explores the digital intelligence technology to strengthen the quality control and evaluation of TCM, and discusses the digital intelligence technology-assisted diagnosis, treatment (the four diagnostic methods of TCM) and disease prevention to inject new productivity into healthcare reform and innovation with digital intelligence technology, bring about a significant change in healthcare, and speed up the entry of TCM into the precise, intelligent and digital era (Duan et al., 2021), summarized as Figure 1.

## 2 Digital intelligence technology and TCM

**FIGURE 2:**
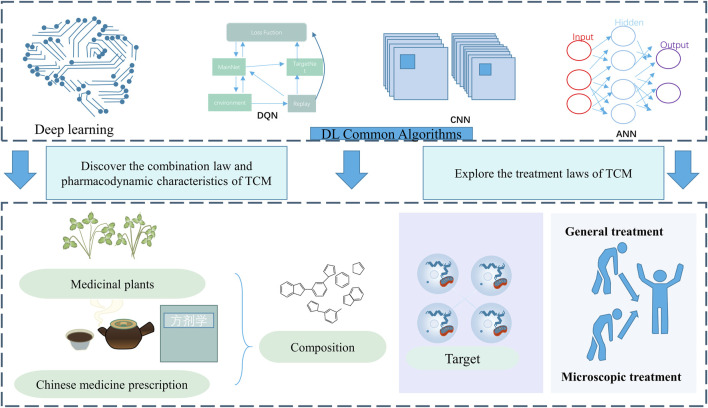
Application of digital intelligence technology in TCM

With the rapid development of digital technologies such as big data, cloud computing, and artificial intelligence, represented by deep learning and large language models, “digital intelligence” has become a new driving force for developing the TCM industry (Duan et al., 2021). Digital Intelligence” has become a new driving force for the development of the TCM industry. In the field of TCM, Digital Intelligence technology mainly refers to the use of big data, cloud computing, artificial intelligence and other technologies, taking TCM big data as an important carrier, digitizing TCM theory, patient information and other data, constructing a variety of artificial intelligence computing models, establishing relationships and collecting a large number of related data to promote TCM research (Fan et al., 2023), as illustrated in Figure 2. The Digital Intelligence technology generates Digital Intelligence within TCM by leveraging extensive digitized data on TCM and patient profiles. This technology integrates the theoretical principles and clinical expertise of TCM with contemporary tools like big data, cloud computing, and artificial intelligence. The aim is to elucidate the precise composition and mechanisms of medicinal effects, enabling accurate prescription, rational formulation, intelligent manufacturing, quality control, clinical precision, and targeted therapeutic outcomes. This approach enhances the research and utilization of traditional TCM practices. This enables the study and application of TCM to be better combined with modern medicine and provides new ideas and methods for the inheritance and innovation of TCM (Guan and Guohua, 2023).

### 2.1 Artificial intelligence and TCM

Artificial Intelligence (AI) is a broad interdisciplinary subject based on computer science, cybernetics, information theory, neuropsychology, philosophy, *etc.* It is a new and developing frontier subject with new ideas, new concepts, new theories and new technologies. AI is based on computer simulation of human intelligence, obtaining information through training, applying or developing relevant algorithms and solving complex problems, usually including natural language processing, expert systems, machine learning and deep learning (Li et al., 2023a).

TCM theory emphasises the importance of the ‘monarch, minister, assistant and messenger’. The relationship between these and the rules of drug combination is of great importance in the treatment of disease. Individualised, fragmented and vague clinical experience and knowledge of TCM make it difficult to explain scientifically. AI can search for hidden information in large amounts of data based on algorithms, discover the rules of drug combinations in TCM, explore the rules of use of TCM, and perform frequency analysis, cluster analysis, *etc.*, To conduct qualitative and quantitative analysis of the compatibility rules, thus reducing research costs and risks.

At present, the research hotspots of AI in the field of TCM are the identification of the quality of Botanical drugs, the study of the effectiveness of formulas and the combination of formulas, and the use of AI-related technologies such as neural networks, data mining, machine learning, feature recognition, image processing and expert systems. The research will focus on tongue diagnosis and syndromes of TCM, exploring the possibility of combining several TCM diagnostic methods from the development of a single diagnostic method (Jieyi et al., 2023). The quality of traditional Chinese botanical drug materials is the basis for the research and development of new TCMs and a guarantee of clinical efficacy. AI techniques such as image processing and computer data mining are gradually being applied to the identification of traditional Chinese botanical pieces. The research and development of new traditional Chinese botanical drug is complicated, diverse, and has a long development cycle. AI can imitate the intelligent activities of the human brain, extract key practical information from a large amount of medical knowledge, construct complex information processing models for predicting research and development directions, and be applied to many aspects, such as drug target discovery, biomarker research, and active metabolite screening. AI-assisted virtual screening technology can be used to screen molecules from multiple TCM databases, dock the selected molecules using various virtual screening methods, and then conduct *in vitro* and *in vivo* experiments after structural modification and ADMET prediction, for the treatment of diseases (Tang et al., 2024).

### 2.2 Machine learning and TCM

At present, the causes of safety issues in TCM production are complex, and the traditional regulatory model has made it difficult to effectively regulate safety issues in TCM production (Zhang et al., 2022b). Any deficiency or inadequacy in any link of the TCM production supply chain may trigger a TCM safety incident. In the field of medicinal plants, the proportion of chemical metabolites is extremely sensitive to external factors. This characteristic increases the complexity of assessing the quality of Chinese medicinal materials. In recent years, with the introduction of new policies in China, TCM innovation has been encouraged and reforms in the review and approval of new drugs have been continuously promoted. However, the current institutional quality management of clinical trials is mainly based on a purely manual model, which is time-consuming, costly and inefficient, and it is difficult to achieve homogeneity in quality management.

Machine learning, as a branch of AI, possesses superior computational and analytic capabilities, improving the overall performance of data fusion, providing reliable problem-solving, and possessing important estimation and predictive capabilities (Cheng et al., 2023). Machine learning algorithms are divided into supervised learning, unsupervised learning, ensemble learning, deep learning, and reinforcement learning. Supervised learning is trained using labelled data to identify the relationship between data characteristics and target variables, and is suitable for classification and regression tasks. Supervised learning for quality attribute detection in TCM preparations can identify the relationship between spectral characteristics and sample quality attributes by analysing labelled data. Classification algorithms such as decision trees, random forests, naive Bayes and K-nearest neighbours are mainly used for qualitative analysis such as species identification and geographical origin. Regression algorithms, including logistic regression, ridge regression, Gaussian process regression, support vector machines and artificial neural networks, are used to predict the content of major metabolites in the sample and are suitable for quantitative analysis of the chemical composition, toxicology and pharmacological activity. TCM data often contains too much complex feature information. Machine learning is used to mine massive amounts of information and discover the efficacy characteristics of TCM, including drug target discovery, TCM quality evaluation, compatibility optimisation, drug action mechanisms and TCM auxiliary diagnosis, effectively shortening the data analysis time (Li X et al., 2022; Zhang and Wang, 2023a). Traditional machine learning can be used to discover the characteristics of Botanical drugs and detect Botanical drugs. In the detection of Botanical drugs, traditional machine learning usually adopts exploration support vector machine (SVM), random forest (RF), K-nearest neighbour (KNN), artificial neural network (ANN), backpropagation (BP), convolutional neural network (CNN) and graphical convolutional neural network (GCN) (Li D et al., 2022), applied to the study of diseases that TCM is effective in addressing various health conditions by examining and understanding the principles of diagnosis and treatment (Zulkifli et al., 2023; Tang et al., 2021) utilized a random forest algorithm, a machine learning technique, to extract features for the study of insomnia diagnosis and treatment within TCM. Their findings demonstrated the efficacy of machine learning in the profound analysis and exploitation of medical record data for TCM’s areas of strength.

Unsupervised learning does not require labels and is designed to discover hidden structures and patterns in data. It is commonly used for clustering and association rule learning. Unsupervised learning algorithms can discover natural groupings or clusters in unlabelled data without predefined category labels. The objectivity of these algorithms provides unbiased insights into TCM research, but they are sensitive to outliers and must be handled with care to ensure accurate pattern recognition (Zhou et al., 2010). Common unsupervised learning methods include principal metabolite analysis, hierarchical cluster analysis, Gustafson-Kessel clustering and fuzzy C-means clustering. They are particularly important when dealing with high-dimensional data and complex datasets, and can reveal deeper levels of information. Reinforcement learning learns optimal decision-making strategies through the interaction between an agent and its environment, focusing on learning from trial and error. Reinforcement learning learns optimal decision strategies through the interaction between an agent and its environment. Its application in TCM is not as widespread as supervised or unsupervised learning. Due to the lack of a definitive answer key and interpretation of the agent’s behaviour, reinforcement learning requires a large amount of data and computational resources, and debugging and interpreting the model is challenging. Although reinforcement learning has made significant progress in some areas, it is still not as widely used as supervised or unsupervised learning algorithms. The complexity of reinforcement learning, the difficulty of understanding and diagnosing agent behaviour, the dependence on large amounts of data and computational resources, and the challenge of debugging and interpreting the results have limited its widespread use compared to other machine learning techniques (Wu et al., 2024).

In the realm of authenticity identification, the integration of spectral analysis technology with advanced machine learning algorithms—such as SVM, RF, and DL models—enables high-precision identification of common medicinal materials like zedoary turmeric and bitter almond (Lin et al., 2022b). This fusion significantly enhances the speed and accuracy of identification processes. In the area of quality tracking and prediction for TCM, multi-modal data fusion technology, which incorporates data from images, mass spectrometry, chromatography, and spectroscopy, allows for tracing the origins of materials such as dried ginger and zedoary. It also enables the prediction of quality change trends in medicinal materials (Zhang et al., 2022c). In chemical composition analysis, machine learning algorithms like SVM and RF have proven highly effective in processing complex chemical data and predicting pharmacodynamics.

Compared to rule-based TCM diagnosis and treatment methods, traditional ML algorithms can learn features through training data and more easily identify the most distinctive features from vast medical records. These algorithms have shown improvements in feature selection and extraction, multi-modal data fusion, and the generalization ability of models. However, they still lack a deep understanding of TCM theory.

### 2.3 Deep learning and TCM

Deep learning is an important branch of machine learning, which is inspired by the study of neural network cells in the human brain. It simulates the way the human brain processes information by building a multilayer neural network model. DL complements the limitations of traditional ML algorithms in dealing with complex relationships, data label imbalance, uncertainty modeling, etc (He and Yong, 2023)*.* DL includes multilayer perceptrons, recurrent neural networks, autoencoders and attention mechanisms. Researchers can help convolutional neural networks get more training samples by generating data enhancements for adversarial networks to improve recognition rates. In addition, existing studies based on DL-binding molecular networks enabled them to identify dopant species not contained in the spectral library. It shows good performance in both detection ability and adulteration identification of traditional Chinese medicine. Deep learning, based on deep neural networks, is often used as a new computational modelling technique to discover the combination rules and pharmacodynamic properties of TCM due to its advantages in data-driven pattern recognition and automatic feature extraction (Wang et al., 2025). Through the backpropagation algorithm, the model calculates the prediction error, adjusts the weight of neurons based on the error, and gradually optimizes the model parameters to achieve optimal performance. Deep learning uses multi-layer neural networks to represent and learn from data, automatically extract features and perform classification or regression tasks. It consists of multiple hidden layers to learn complex non-linear relationships and automatically extract high-level features, and typically requires a large amount of training data and powerful computing power. Compared to traditional machine learning methods, deep learning can better extract feature information from data and exploit the interactions and differences between these features to better adapt to the complexity of the model. Artificial Neural Networks (ANN) and Convolutional Neural Networks (CNN) are the two main deep learning architectures (Li et al., 2024). ANN improves generalisation and fault tolerance by adjusting weights, while CNN extracts feature information through convolutional layers, although it may face the problem of gradient disappearance or explosion. The Residual Convolutional Neural Network solves this problem by introducing a residual module and skip connections, which improves the efficiency of data analysis.

**FIGURE 3:**
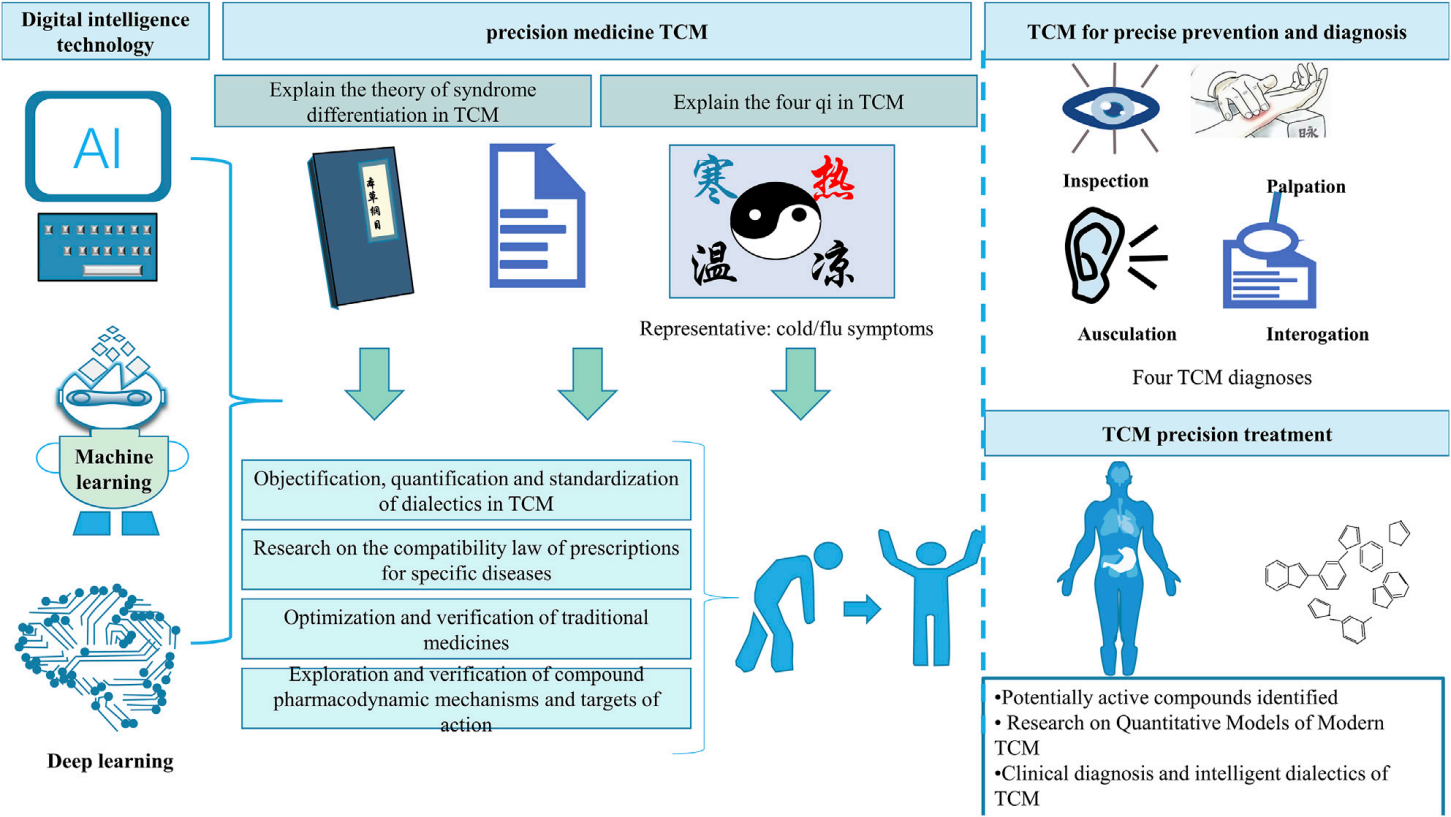
Deep learning and TCM.

In recent years, a growing number of Chinese researchers have initiated the application of neural networks to delve into the therapeutic principles of TCM, thereby substantiating its objective efficacy (Huang et al., 2022). In the face of the imbalanced dataset of rare evidence types and model diseases in TCM dialectics, neural networks may encounter the problem of overfitting (Li et al., 2023b). Researchers initiate their investigation by focusing on evidence types, which are then subdivided into finer evidence-type factors. Utilizing deep learning techniques within a real-world model, they use the clinical efficacy of TCM as the outcome variable to elucidate the intricate connections among formulas, herbal medications, metabolite metabolites, molecular targets, and phenotypic outcomes (Pan et al., 2023), as illustrated in Figure 3. Deep learning techniques can also analyse the chemical composition of TCM to distinguish its geographical origin, age and medicinal parts. The imaging of HPLC fingerprint data and the application of ‘binary multiple information fingerprinting’ technology further improve the classification accuracy of the model. These studies have shown that deep learning can effectively extract chemical fingerprint information, reduce data pre-processing and feature selection steps, and improve model accuracy. However, the interpretability of features extracted by deep learning remains a challenge and requires further research.

## 3 Digital intelligence technology enhances quality control and evaluation of TCM

The quality of Botanical drugs is the foundation of clinical efficacy and the fundamental of establishing the value system for TCM (Zhang et al., 2023a). The quality of TCM is the overall performance of the comprehensive biological effects of the chemical substances of TCM, and its inherent characteristics are multi-component, multi-efficacy and integrity. There are many factors that affect the quality of TCM: medicinal material varieties, cultivation and ecological conditions of origin, harvesting and processing, preparation technology, transportation and storage, extraction and purification, preparation technology, formulation, and drug metabolism (Cheng and Lianxin, 2023). It is the core of the value system of TCM. The technology system for evaluating the quality of Botancial drugs emphasizes the TCM attributes of “appearance, colour, aroma, and flavour.” It aims to analyze these characteristics to determine the authenticity and quality of TCM products (Liu and Xueb, 2022). The contemporary quality assessment framework for TCM is grounded in the research paradigm of “identifying metabolites and quantifying metabolites.” This system primarily encompasses chemical and biological evaluations (Li et al., 2023c). Chemical evaluation is founded upon high-performance liquid chromatography (HPLC), gas chromatography (GC), liquid chromatography-mass spectrometry (LC-MS), near/mid-infrared spectroscopy (NMIR) and other chromatographic and spectroscopic techniques (Zhang and Wang, 2023b). There are many kinds of chemical components and complex structures of Chinese medicinal materials. While this feature provides a material basis for the efficacy of TCM, it also brings many challenges to the research on the quality standards of TCM. With its advantages of high sensitivity and high resolution, mass spectrometry can quickly and accurately identify biologically active compounds in TCM compounds, and provides strong technical support for the establishment of TCM quality standards. In recent years, the combined technology of chromatography and mass spectrometry (such as high performance liquid chromatography-mass spectrometry combined technology HPLC/MS and gas chromatography-mass spectrometry combined technology GC/MS) has been widely used in the research of TCM. These technologies combine the efficient separation capabilities of chromatography and the high-sensitivity detection capabilities of mass spectrometry to more accurately identify and quantify biologically active compounds in TCM. For example, HPLC/MS is widely used in the identification of flavonoids in TCM compounds because of its efficient separation of complex samples and high-sensitivity detection of polar compounds; GC/MS is commonly used in the analysis of volatile oil components of TCM due to its good separation and detection performance of volatile components. The liquid-mass combination technology combines the high separation performance of chromatography with the high identification ability of mass spectrometry to realize the complementary advantages of the two. In the field of TCM analysis, liquid-mass combined technology, with its advantages of high efficiency, high sensitivity, simple sample processing, and low dosage, has greatly improved the efficiency and accuracy of TCM analysis, and realized the automation of TCM analysis (Li et al., 2022i). The development of electronic sensory technologies such as electronic nose, electronic eye, and electronic tongue has enabled the system to realize objective data expression of some traits of medicinal materials or decoctions. The introduction of big data and AI technology has provided a new opportunity for in-depth interpretation of the chemical characteristic information of TCM and the realization of the identification of TCM based on chemical characteristics. Through the collection of a large number of samples of comprehensive chemical quantitative information, the use of AI technology to extract high-dimensional chemical information laws, remove the interference of various noise information, and discover the “chemical characteristics” (composition, magnitude, and relative proportional relationship between components, *etc.*) that determine the variety, and then realize the quality integration of TCM based on chemical characteristics. Identification, reflecting the quality transmission process of TCM and realizing the traceability of quality information, provides key support for the construction of a quality control system for the whole process of TCM.

**FIGURE 4:**
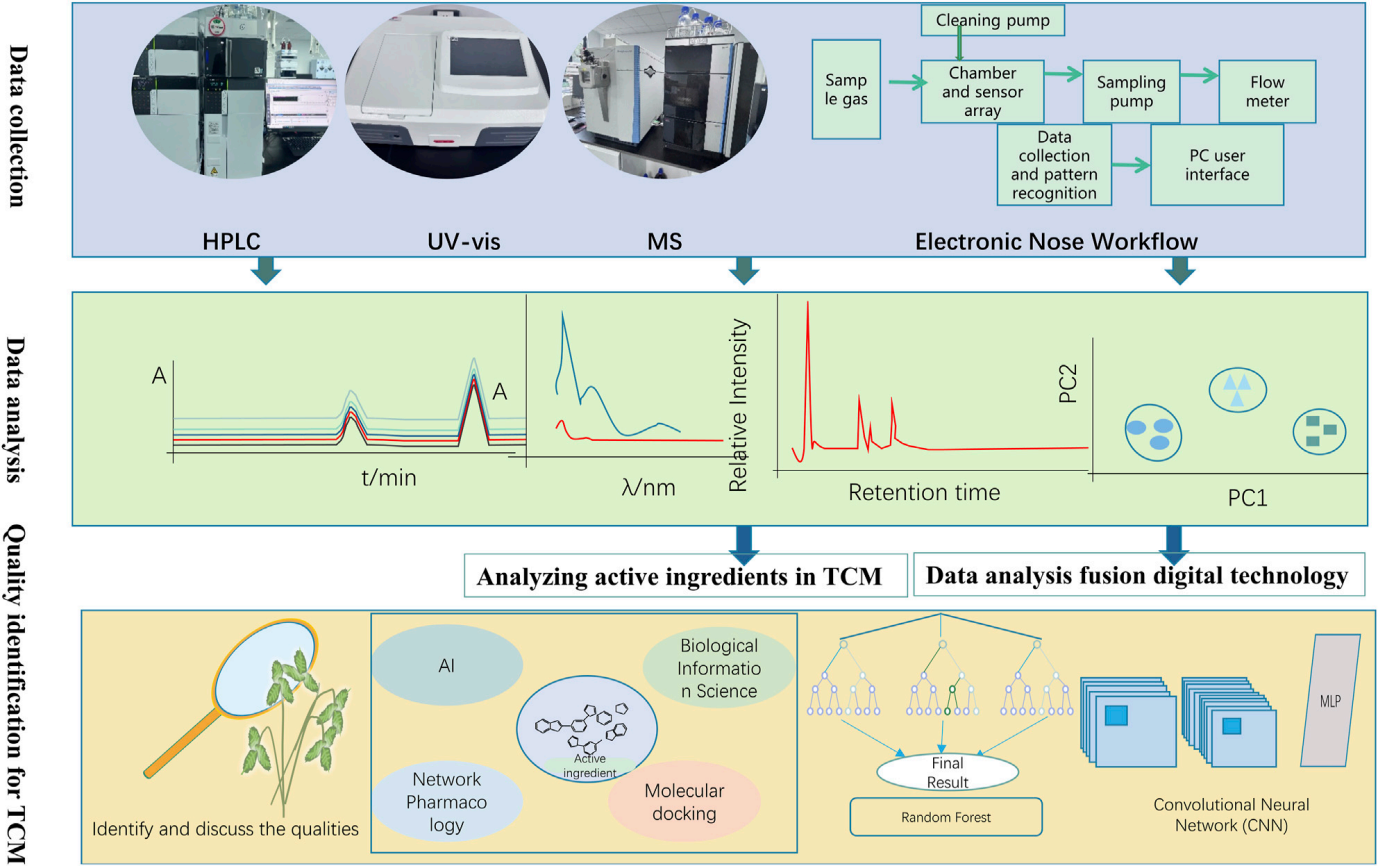
Digital intelligence technology enables “precision TCM” under the Premise of great health.

In recent years, Chinese scholars have adopted the Q-marker approach to regulate the quality of TCM. It serves as a quality marker specific to individual TCM monomers, integrating the synthesis of TCM metabolites and their origin pathways, macroscopically representing the complex material metabolites and intrinsic quality characteristics of TCM. By doing so, It can ensure the clinical efficacy of TCM and enhance the consistency and control over the quality of TCM (Bai et al., 2018). The quality of Botanical drugs is consistent and controllable. Influenced by soil, climate, topography and other factors, there are differences in the content of active metabolites in the same Botanical drug. A single analytical technique cannot fully represent the active metabolites of the Botancial drug, which brings difficulties to the quality evaluation of the Botancial drug. Numerical Intelligence technology can be used to mine the information on various Botanical drugs, realize automatic identification of Chinese herbal medicinal tablets (Miao et al., 2023) and establish an ideal resource database (Khan et al., 2021; Wang et al., 2022c). It can also be used to build a scientific and convenient evaluation method of “identifying the symptoms and evaluating the quality” (Xu et al., 2023), as illustrated in Figure 4. In the research of (Yang et al., 2020), by evaluating the effects of Botancial drugs, they constructed the Keras framework based on deep learning, aiming to achieve rapid and intelligent identification of Botancial drugs. Utilizing the maximum entropy model and statistical analytical techniques, the researchers integrated data about ecological factors, the geographic distribution of Astragalus, and the content of crucial indicator metabolites. Their objective was to predict the spatial distribution of environmental suitability for Astragalus, elucidate the correlations between Astragalus methyl glycoside and mulligatranin isoflavone-7-glucoside with ecological factors, and delineate the suitability zones for high-quality Mongolian Astragalus within the Inner Mongolia region of China (Yang et al., 2020). The data were used to predict the environmental suitability distribution of Astragalus.

TCM decoctions are extensively employed in clinical practice. The integrity of their storage is paramount for ensuring both the therapeutic efficacy and the safety of patient administration. The conventional manufacturing process for Xiao Chai Hu (XCH) capsules in TCM is characterized by its time-intensive, labour-intensive, and expensive nature. In response, a novel fluidized bed granulation and drying technique, employing water as the binder, has been developed and refined following the Quality by Design (QbD) philosophy Teng et al. (2023), Chen (2023) observed a real-time spectral analysis of the storage quality of Sijunzi Tang decoction based on a robotic operating system (ROS) (Guo et al., 2021). utilized a deep learning-assisted mass defect filter (MDF) in conjunction with high-performance liquid chromatography quadrupole time-of-flight tandem mass spectrometry (UHPLC-Q-TOF/MS) to facilitate rapid classification and precise assessment of the quality of Living Blood and Sheng Shi Tang.

### 3.1 Spectroscopy

Ultraviolet-visible (UV-Vis), Infrared (IR), and Nuclear magnetic resonance (NMR) are extensively employed as qualitative tools to obtain diverse chemical information in the quality assessment of Botanical drugs, summarized in Table 1. In the big data environment represented by deep learning and integrated learning, new machine learning algorithms have begun to be applied to the quantitative and qualitative modelling of NIR spectra. However, they encounter issues such as network size, optimal parameter selection, overfitting, and model interpretability. How to better combine deep learning algorithms for machine learning with spectral analysis is of increasing interest to researchers (Ding et al., 2023; Pan et al., 2023). (Ding and Sihai, 2023) investigated a novel approach for distinguishing Gentiana herbs of various origins by utilizing a deep forest model integrated with spectroscopy. Their study focused on Gentiana herbs sourced from diverse regions within Gansu province (Jin, 2024). used Raman spectroscopy combined with machine learning methods to classify and identify the easily confused mineral Chinese and Mongolian medicinal herbs. Zhang et al., (2023b) studied SERS Raman spectroscopy combined with the SSA-BP neural network to identify colouring adulteration in Dendrobium, Saffron and Turmeric rapidly.

**TABLE 1:** Advantages and disadvantages of common spectra in the detection of Botancial drugs.

Spectroscopy	Application features	Advantage	Disadvantage
NIR	• Multiple and combined frequency absorption of the vibrational spectra of C-H, O-H, N-H and other hydrogen-containing groups of atoms within a molecule • Qualitative analysis of samples, mostly used for quantitative analysis • Identification of the authenticity of Botancial drugs • Quantitative analysis of active metabolites in TCM • On-line testing of Chinese medicine preparations	• Efficient, fast and cost-effective • Non-destructive testing of solid, liquid and powdered samples • Infrared fingerprinting is highly specific, reproducible, easy to operate, and can provide rich molecular information • Infrared spectroscopy can systematically and comprehensively respond to the differences in the chemical composition of Botanical drugs in different species, different origins and different preparation methods	• There are differences in the extraction of spectral information by different methods • A large number of samples taken • Data processing is more complex • Relatively poor reproducibility between instruments • Weak model generalization
NMR	• 1H NMR spectroscopy is the most commonly used method for the identification of botanical Chinese medicines • Used in the development and identification of new drugs, and the determination of the type and content of characteristic chemical substances	• Simple and convenient, non-invasive, dynamic • The 1H NMR method provides a more accurate method for the rapid identification of authentic Chinese medicines • 1H NMR profiles are singular, comprehensive, quantitative and easily recognizable	• signal overlap problem • Long collection time and high sample volume • High cost and maintenance • Lower sensitivity
Raman	• Mainly used in the identification of TCM, content determination, spectral attribution of metabolites, detection of pesticide residues, detection of illegal additives, research on authenticity, concocting process research, quality change researchetc. • Raman scattering is a method of analyzing Raman spectra formed by analyzing information such as changes in the intensity of scattered light with energy (or frequency) and changes in the polarization of the scattered light relative to the polarization of the incident light, and then obtaining information on the vibrational and rotational energy levels of molecules, and then analyzing metabolites qualitatively and quantitatively	• No chemical, mechanical or photochemical decomposition of the sample • Realization of non-destructive testing • Qualitative and quantitative analytical methods with non-contaminating processes • Rapid detection • Accuracy of results • high sensitivity • The small sample size required and simple or no sample handling • Advantages such as low cost of testing and unaffected by water	• Poor reproducibility and stability of the active substrate of the SERS method • The specificity of the active substrate of the SERS method leads to high costs and precludes mass production • When the metabolites to be measured are too complex, the same groups of different substances may appear to be superimposed on the peaks, and then it is necessary to rely on complex and time-consuming pre-treatment techniques such as TLC + SERS, UPLC + SERS and MIP + SERS. • There is no complete theory that can explain all SERS effects
UV-vis	• Measurement of the absorption of light at wavelengths from the ultraviolet to the visible • Qualitative analysis of Chinese medicines for authenticity identification • Different origins of Botanical drugs, the origin of identification •	• Suitable for qualitative and quantitative analysis of a wide range of metabolites • simple principle • easy handling • Wide range of uses • high sensitivity • High accuracy	• May require complex sample pre-treatment • Lower sensitivity for some metabolites • The extraction of the herbs with solvents resulted in broad absorption peaks and serious overlap in the UV spectra of the extracts • The UV spectral absorption of herbs with high similarity is similar, which may lead to inaccurate identification of the herbs

### 3.2 Chromatography

Chromatography has become a significant technique in analyzing Botanical drugs due to its powerful separation ability, but it requires expensive equipment and time, summarized as in Table 2. The current research focus is on the development of an online comprehensive two-dimensional liquid chromatography system, enhanced by AI, to thoroughly elucidate the variability among different morphological species of herbal medicines and to authenticate the integrity of herbal remedies Du et al. (2023), Huang (2024) established a framework named MCnebuIa (MuItipIe-CliemicalNebula), which facilitates the analysis of medicinal plant mass spectrometry data by focusing on crucial chemical classes and multi-dimensional visualization Shi et al. (2024) employed rapid evaporation ionization mass spectrometry (REIMS) fingerprints in conjunction with machine learning to quantify the distinct species-specific variations in the REIMS profiles of various morphologies of Phellodendron amurense from Hubei Province. The method effectively predicted and differentiated the species of the analyzed samples Shi et al. (2023) investigated the application of dry REIMS technology, integrated with machine learning, for the precise discrimination of Baektuwang and its common imitations, including Korean Baektuwang and Dahuocao.

**TABLE 2:** Advantages and disadvantages of common chromatograms for quality testing of Botanical drugs.

Chromatography type	Application features	Advantage	Disadvantage
• HPLC	• A key method for drug analysis and testing in Botancial drug, covering identification, impurity control, content determination, and combating counterfeits	• High-resolution analysis • Highly repeatable • Chromatographic columns are reusable • Automated operations • Multidimensional analysis • Suitable for analysis of a wide range of metabolites	• High solvent costs • Complicated operation • Detector limitations • Combined separation of readily decomposable samples • Less versatile
• UHPLC	• Identification of metabolites in Chinese medicinal preparations by ultra-performance liquid chromatography • Commonly used for quality analysis of Botanical drugs	• Fast analysis • Good separation • Test accuracy • Reduction of matrix effects	• High equipment costs • complicated operation • High demand for samples • Some metabolites are not applicable • Not suitable for high-volume analysis • Solvent costs and environmental pollution • Lack of universal detectors
• GC	• TCM’s volatile odour metabolites are best detected and separated using gas chromatography	• High sensitivity and selectivity • Fast detection data • Determination of moisture content • High sensitivity and selectivity	• Not suitable for the analysis of difficult-to-volatilize and unstable substances
• HPLC-MS	• Identification and Content Determination of TCM • To determine the authenticity and quality of TCM	• Identification of biologically active compounds in TCM • Strong selectivity, high sensitivity, good separation ability, low sample loss	• Expensive instruments • Bulky • The operation is relatively complex • High equipment cost
• GC-MS	• Suitable for identification and quantification of volatile components	• Analysis of volatile oil components and their biological activities in TCM • Suitable for identification and quantification of volatile components	• Not applicable to thermally unstable compounds • Bulky • The operation is relatively complex • High equipment cost

### 3.3 AI sensing technology

AI sensory technology can replicate human senses, enabling rapid acquisition of the shape, colour, and scent of Botanical drugs. Pattern recognition can assess the quality of these medicines by providing a comprehensive display of their characteristics. This technology objectively quantifies the subjective criteria of “shape, colour, and scent” used in evaluating the quality of Botanical drugs, transforming them into data-driven representations, summarized in Table 3.

**TABLE 3:** Characteristics and advantages and disadvantages of the application of electronic sensory technology.

Electronic sensory technology	Application features	Advantage	Disadvantage
• Electronic eye	• Simulation of human vision, analysis of sample colour, colour distribution and other visual parameters	• Simple, fast, no pre-treatment; suitable for quality identification and evaluation of TCMs	• Most of the Chinese medicines need to be observed after crushing, which destroys the integrity of the Chinese medicines; the image recognition and analysis functions need to be improved to realize non-destructive observation
• Electronic nose	• Mimics human odour recognition, consisting of sensor arrays, signal processing systems, and pattern discrimination systems	• Simple, non-destructive sample preparation and relatively rapid evaluation of the assay; high sensitivity and selectivity for the odour sample being measured	• High requirements for the surrounding environment, stability is not high; mostly foreign imported equipment, proprietary, specificity is poor, need to develop sensor arrays suitable for TCM research
• Electronic tongue	• Non-specific, low selectivity, high stability, cross-sensitive chemosensor arrays for different samples in solution	• Good reproducibility, standardizable control, fast detection response; applicable to quality identification and evaluation of TCMs	• Most of them are imported from abroad, with poor proprietary and specificity, and it is necessary to develop sensor arrays applicable to the research of TCM.

The development of advanced sensory information fusion technology, utilizing multi-source information fusion techniques, is derived from AI. This technology aims to efficiently and accurately identify specific attributes of the subject under study. By integrating various types of sensory information in a complementary manner, this approach enhances the comprehensiveness and precision of the information gathered Li et al. (2023), Ma et al. (2024) used an electronic tongue and electronic eye combined with a deep learning model to collect gustatory fingerprint information and visual image information of different mussel varieties to identify mussel varieties rapidly (Ren et al., 2024). used an electronic nose and electronic tongue to evaluate the odour and taste characteristics of 11 batches of Panax pseudoginseng samples from different origins to identify Panax pseudoginseng from various origins (Wang et al., 2024). employed the electronic nose technology and gas chromatography-ion mobility spectrometry (GC-IMS) technology to detect the volatile differences between northern and southern Panax quinquefolium. They utilized the NIST and IMS databases for qualitative and quantitative analysis of volatiles and conducted the Dynamic Principal Metabolites Analysis (DPCA) on northern and southern Panax quinquefolium samples. DPCA was used to analyze the characteristic substances of North and South Chaihu and screen the volatile substances as markers of odour differences (Yang et al., 2024). used electronic tongue, electronic nose and gas chromatography-ion mobility spectrometry (GC-IMS) to compare the taste, odour and volatile organic metabolites among different basal rhubarb tablets to carry out the identification study and to establish the basal discriminatory standards. Digital Intelligence Technology Helps to Achieve Precision in Botanical Drugs in Table 4.

**TABLE 4:** Mathematical Intelligence Technology Helps to Achieve Precision in Botancial drug.

AI algorithms	Key technology	Quality evaluation models for botancial drugs	Sketch	Bibliography
machine learning	SVM, RF, PCA, NBC, KNN, CV-tech	TCM quality evaluation model	• Extraction of colour, speckle, texture and geometrical features of TCM slice images • Identify the quality of Chinese herbal medicinal tablets	Gao et al. (2024)
	SVM, PCA, OPLS-DA, HPLC, UV-Vis, Network Pharmacology、		• Establishment of quality indicators related to key factors affecting liquorice quality • Glycyrrhizin, glycyrrhizin, glycyrrhizic acid and glycyrrhizin content are vital indicators	Chen et al. (2023)
	CNN、SVM、RF、MLP	1D-CNN model	• Best bitter flavour discrimination based on Raman spectroscopy	Cheng et al. (2023)
	PCA、SVM、LDA	colour discrimination model	• Establishment of colour parameters for dried ginger and its ginger charcoal artefacts at different levels of preparation • Predicting changes in chemical composition content	Zhang et al. (2022c)
	PLS-DA、SVM、LDA		• Rapid mass analysis of Curcuma longa and Curcuma vinifera • Determining the degree of quality control and concoction of Curcuma longa drinking tablets	Lin et al., (2022b)
	RF		• Inferring trends in the chemical composition of bitter almonds • Colour-odour fusion information measurement to assess the bitter almond quality	Ouyang et al. (2023)
machine learning	CNN	Public Database of TCM Images	• Image Recognition and Retrieval of Herbal Medicines	Sun and Qian (2016)
machine learning	CNN	Herbal knowledge mapping model	• Constructing Herbal Knowledge Graphs and Proposing Graph Convolution Models for Multi-Layer Information Fusion • Obtain informative characterization of symptoms and herbal features	Yang et al. (2022b)
machine learning and AI Sensory Technology	PCA、SVM	multi-source information fusion technology	• Exploring the two classifications of the five flavours for identification of medicinal properties	Ren et al. (2023a)

## 4 Digital intelligence technology and precision medicine in TCM

At present, the clinical services of TCM in our country are still based on the traditional dialectical treatment model. The diagnosis and treatment process is greatly affected by the thinking habits of doctors and their personal professional level, making it difficult to guarantee the consistency of dialectics. In the diagnosis process of TCM, the collection of information from the four consultations (observation, olfaction, inquiry, and palpation) is the key link to realize the diagnosis of the disease, which can transform the patient’s vague symptoms and signs into accurate symptomatic diagnosis. However, the real clinical manifestations of patients are complex and diverse, and even if it is the same disease, the diagnosis process is difficult to mechanically complete the collection of all four diagnosis information according to a unified template. With the help of interdisciplinary and interdisciplinary methods such as biology, mathematics, and artificial intelligence, the digital characterization of the status of disease symptoms can be studied in depth, so as to enhance the digital level of dialectical treatment of TCM, enhance the understanding of the evolution of the symptoms of dominant diseases, and construct a new model of combined disease and symptom research. By integrating the digital information collected from the four consultations into a clear symptomatic diagnosis and intelligently associating corresponding treatment measures, it is expected to realize the precision and standardization of TCM diagnosis and treatment. The collection and analysis of diagnosis and treatment information in TCM is highly subjective, which makes it difficult to guarantee the reliability, accuracy and consistency of diagnosis and treatment information. In the combined diagnosis system of disease and symptoms, the individualized characteristics of patients further increase the complexity of information measurement, making it difficult to form a standardized and unified information template. In addition, in the process of continuous progress of chronic diseases, the characteristics of the disease are complex and changeable, and precise prevention and treatment is facing greater challenges.

**FIGURE 5:**
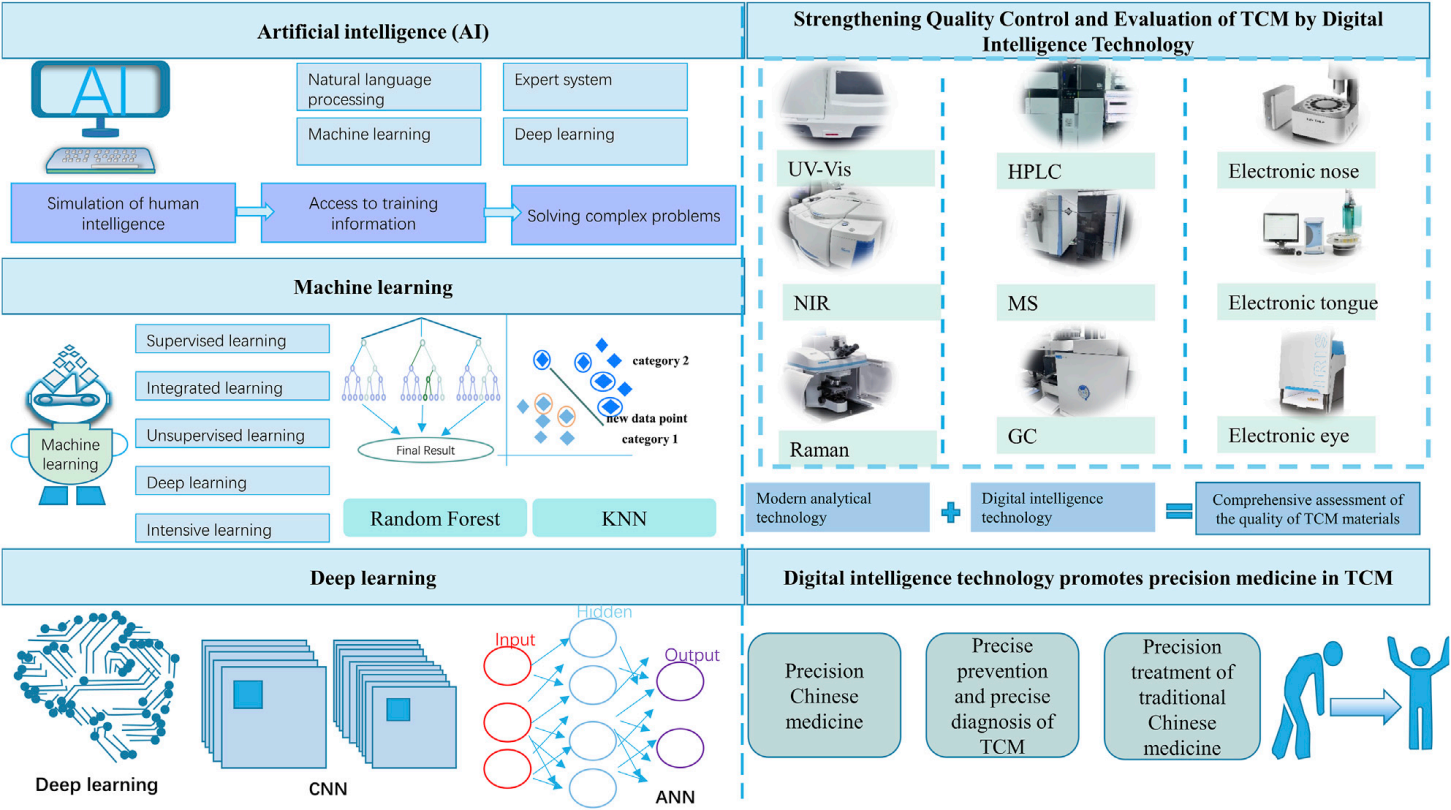
Digital intelligence and precision medicine in TCM

Since 2003, the Chinese government has initiated several scientific programmes for the precision of TCM, one of which is the digitization of ancient documentary data, clinical data and research publications in TCM, and another is the design and screening of rich molecular metabolites contained in TCM prescriptions as a potential resource for new drugs (Zhu et al., 2022). The second is designing and screening rich molecular metabolites in TCM prescriptions as potential resources for new medicines. With the rapid progress in genome sequencing technology and the integration of bioinformatics with big data analytics, precision medicine builds upon the principles of personalized medicine to develop preventive and therapeutic strategies tailored to individual patient characteristics (Chen et al., 2022), which provides a new direction for the development of modern TCM research, as illustrated in Figure 5.

### 4.1 Digital intelligence technology for precision medicine in TCM

TCM has been an individualized treatment since ancient times, and to a certain extent, it aligns with the contemporary concept of precision medicine. The identification and treatment of TCM entail a highly individualized process grounded in an in-depth understanding of unique patient symptoms and attributes. Precision medicine in TCM integrates the principles of TCM theory with advanced precision medicine technologies and patient diagnostic data from the four diagnostic methods. This approach aims for precise disease categorization, diagnosis, and treatment, emphasizing individualized, holistic, process-oriented, and comprehensive strategies for disease prevention and clinical management (Chen et al., 2016; Jia, 2017). TCM, as a unique medical treatment model in China, has similarities with the internal logic of AI in that its theoretical system of a holistic view, view of change, and thinking mode that emphasises deduction and intuitive understanding based on accumulated experience share common ground with AI’s emphasis on the holistic effect, open dynamics, experiential thinking and predictive reasoning. The focus is on individualized, systematic, whole process, whole element and global disease prevention and clinical treatment. Advances in computing technology, including deep learning and fundamental models, have pushed the exploration and modelling of complex systems to a new stage (Fan et al., 2023).

The convergence between heaven and earth and its consistency is the core connotation of precision medicine in TCM (Huang et al., 2021). Modern computer technology offers a transformative avenue for the innovation and advancement of TCM. This encompasses research aimed at establishing standardized frameworks for TCM, the application of AI algorithms in TCM practices, and the exploration of advanced TCM diagnostic and therapeutic models (Chen et al., 2006; Liu, 2023). Utilizing data mining AI technology and mathematical principles to investigate the theoretical foundation of TCM, scholarly perspectives of renowned practitioners, TCM diagnostic and therapeutic approaches, clinical technologies in TCM, prescription models in TCM, and clinical effectiveness in TCM. The objective is to facilitate the interdisciplinary fusion of internet medical knowledge, multi-path diagnosis and treatment, and enhance the efficacy of diagnosis, treatment, and management (He et al., 2022b; Huang et al., 2022) based on convolutional neural network cross-feature, establish and validate the classification model of TCM and achieve the dialectical classification of different diseases in TCM (Zhang et al., 2022a). Based on Transformermodel’s neural network model, they developed an auxiliary tool for predicting inpatient prescriptions based on patients’ clinical electronic medical records to overcome the overfitting problem and improve the accuracy rate.

#### 4.1.1 Numerical intelligence techniques to explain TCM evidence theory

Evidence is the cornerstone of the theoretical system of TCM and the core of its diagnosis treatment and clinical diagnosis. Diagnosis involves the integrated analysis of the patient’s clinical data, grasping the current location and nature of the disease, and the thought process of forming a complete diagnosis. TCM diagnosis often has the disadvantages of lack of objectification, quantification and standardisation, the influence of subjectivity in diagnosis and treatment, and its uncertainty, instability and ambiguity are highlighted. This means that patients with the same disease often have different diagnostic results. The lack of standardisation severely limits the systematic research and compilation of TCM. The pressing inquiries in the contemporary endeavour to modernize TCM pertain to elucidating the core nature of TCM evidence, uncovering its scientific essence, establishing the inter-scale relationship between the macro- and micro-aspects of TCM evidence, and formulating a standardized, objective definition of TCM evidence (H. Z. Cheng Lei; Fu ee al, 2023). Due to the medicinal properties of TCM, the sum of biological effects of TCM, its specificity and complexity, it is impractical to use the traditional single-target, single-metabolite research paradigm for the study of TCM, and the use of numerical intelligence technology can better solve the scientific problems in the field of TCM (Zhongquan et al., 2022). Machine learning explores the hidden “disease-syndrome-symptom” associations in complex data based on massive clinical information and a wealth of computational methods, effectively overcoming the subjectivity and limitations of human syndrome differentiation thinking and promoting the standardisation and normalisation of TCM syndrome research. Developing syndrome differentiation models for related diseases, can not only become a powerful tool for doctors to make precise clinical syndrome differentiation decisions but also provide useful ideas for exploring the nature of TCM syndromes (Yeh et al., 2020). applied a cost-sensitive graphical convolutional neural network model to discover the connection between TCM and its meridians using local and global topological features of metabolites to explain TCM theories (Wang et al., 2019). used a machine-learning approach to determine the molecular features of active metabolites in 646 TCMs to provide new evidence for understanding meridian molecules (Xia et al., 2020). evaluated prescription patterns and medication rules through association rule learning, cluster analysis and complex network analysis to identify the TCM kidney disease mechanism as deficiency and damp-heat (Hu et al., 2019). demonstrated the feasibility of using an end-to-end text categorization algorithm to distinguish between Yin and Yang deficiency in unstructured medical records based on the classical convolutional neural network (CNN) and fastText algorithm.

It should be pointed out that there are still some problems to be solved in the research of symptoms of TCM with digital intelligence technology (Yang et al., 2013). Dialectics in TCM is based on the results of the synthesis of information obtained from looking, listening, asking and consulting. However, most symptom models currently focus on clinical symptoms, and it is difficult to comprehensively and objectively reflect the patient’s current symptoms. How to systematically integrate these multimodal data, including tactile, visual, auditory and descriptive text, for modelling has become one of the hot topics in the field of TCM. This involves dimensional reduction of high-dimensional data, objective image extraction, and multi-dimensional data fusion, and must fully consider multimodal complementarity, redundancy, and mutual exclusion to achieve the goal of truly accurate and intelligent dialectics. In the pursuit of model performance, deep learning not only brings greater computational overhead but also inevitably weakens the interpretability of the model. In particular, the “micro-dialectical” research that combines modern indicators makes it difficult to clarify the correlation between clinical symptoms and types from the perspective of TCM. The unexplainability of some output conclusions also severely limits the popularisation and application of the model in clinical practice.

#### 4.1.2 Numerical intelligence techniques to explain the four qi in TCM

With the introduction of methods and concepts such as data mining technology, network pharmacology and machine learning, the field of research on the compatibility rules of TCM formulas has also been continuously expanded, mainly including research on the compatibility rules of formulas for specific diseases, optimisation and verification of traditional medicines, exploration and verification of the mechanism of action and target of action of metabolite medicines, *etc.* Among these, research on the compatibility rules of formulas for specific diseases is the most common. Among these, research on the compatibility rules of formulas for specific diseases is the most common (Li Y et al., 2022). This type of research often applies various data mining techniques and combinations, such as association rules, cluster analysis and hidden structures, to deeply analyse the medication rules for specific diseases.

The four qi of TCM (cold, heat, warmth and coolness) are the core of TCM theory and an essential basis for guiding the clinical prescription. Due to its specificity and complexity, the single-target, single-drug research model is unsuitable for the overall study of the four qi of TCM. Digital intelligence technology can make up for its shortcomings (Ge et al., 2022; Song et al., 2021; Zhongquan et al., 2022) used machine learning (ML) to clarify the “hot and cold properties” of TCM medicine at the molecular level of metabolites with modern scientific connotations (Jia et al., 2023). based on the K-nearest neighbour algorithm to predict the medicinal properties of Chinese herbal metabolites in terms of heat and cold and verified that the structure of Chinese herbal metabolites determines the medicinal properties of Chinese herbal metabolites (Zhang, 2023a). investigated the relationship between the material metabolites of TCM and their cold-heat medicinal properties by constructing a recognition model and proved that TCM with similar cold-heat medicinal properties have similar material bases.

In recent studies, the properties of medicines (functional classification, the four Qi, the five flavours, and the channels they enter) have gradually been incorporated into the algorithm, and relevant weights have been assigned for comprehensive analysis so that the research is more in line with TCM theory. However, the association rules algorithm, which is usually based on large samples, generates a large number of associations. Coupled with the relatively single and formulaic analysis process, the specificity and interpretability of the data mining results are insufficient. Although it is not unrelated to the complex and changeable pathogenesis of clinical diseases and the balance between cold and warm medicines, most discussions based on the analysis results therefore revolve around the five elements, viscera, yin and yang, cold and heat, which are far-fetched and have limited practical significance for clinical guidance (Gu et al., 2023).

### 4.2 Precision prevention and precision diagnosis of TCM under digital intelligence technology

“Preventing illness before it occurs” is the foundational principle of TCM health theory, which underscores the importance of preemptive actions aimed at bolstering the body’s resistance and averting disease (Bentivegna et al., 2023; Wang, 2023) developed the first stroke risk prediction tool with TCM characteristics to improve the primary prevention system of stroke (Zheng et al., 2023). utilized a combination of machine learning algorithms to create a novel stroke risk prediction tool imbued with TCM attributes. This innovative approach resulted in the development of an intelligent prediction application for metabolic syndrome, designed for self-diagnosis by lay users to facilitate early detection and intervention of metabolic syndrome (Tang et al., 2022). developed a predictive model for the recurrence and metastasis of colorectal cancer (CRC) using machine learning techniques and TCM (TCM) factors. This model offers valuable insights for potential implementation in clinical settings (Chen et al., 2024). proposed a holistic TCM diagnosis model based on deep learning for the classification and prediction of multiple TCM symptoms, which aligns with the diagnostic characteristics of holistic TCM (Chien et al., 2021). employed AI algorithms—such as logistic regression, Bayesian networks, and decision trees—to develop an association model that connects metabolic syndrome with TCM constitution. This model serves as a guide for therapeutic, dietary, and care interventions. Gao et al. (2024) utilized machine learning techniques to forecast the onset, progression, and detection of copper deficiency in individuals with active ulcerative colitis. They pinpointed several commonly used TCMs with high frequency, including Atractylodes macrocephala, Salvia miltiorrhiza, Curcuma longa, and Liang and Wang (2023) identified potential biomarkers of ulcerative colitis (UC), predicted target TCMs, and performed early intervention with TCMs, which is essential for the early diagnosis and treatment of colorectal cancer. Zheng et al. (2023) developed a predictive model for metabolic syndrome using Bayesian optimization and XGBoost algorithms, incorporating TCM features to address angiogenic diseases in prevention and treatment.

The diagnostic process in TCM relies on four essential methods: observation, olfaction, inquiry, and palpation, with pulse-taking and tongue examination being frequently utilized techniques (Tian et al., 2024). TCM posits that the tongue acts as a reflective indicator of the internal organs. Alterations in the tongue’s morphology offer objective clues to an individual’s health condition, facilitating evidence gathering, the formulation of therapeutic approaches, medication prescription, and prognosis assessment (Liu Q et al., 2023). Traditional tongue diagnosis is affected by factors such as the external environment and the subjective clinical experience of doctors. Intelligent tongue diagnosis is essential for self-supervision methods, multimodal information fusion, and tongue pathology research for research with tongue diagnosis (Tania et al., 2019). An increasing number of clinicians are progressively embracing computerized tongue diagnosis systems as a valuable medical tool for health evaluation and diagnostic purposes, as illustrated in Table 5.

**TABLE 5:** Application of Smart TCM in tongue diagnosis.

AI algorithm	Key technology	Tongue diagnosis evaluation model	Findings	Bibliography
Machine learning	CNN	A classification framework for tongue features A classification framework for tongue features	• Analysis of extracted features from panoramic tongue images for diabetes diagnosis	Balasubramaniyan et al. (2022)
	SVM、RF、GBDT、DT、		• Modelling automatic, efficient and accurate quality control of tongue images	Jiang et al. (2021)
	CNN		• Classification of tongue texture and tongue coating features to reduce diagnostic variation and make accurate diagnoses	Li J et al. (2022)
Deep learning	CNN、Weakly Supervised Learning、MIL		• Classification of teeth markings on the tongue, detection of tooth-marked areas	Zhou et al. (2022)
	CNN		• CNN recognition of tooth labelling tongue • Computer-aided tongue diagnosis	Wang et al. (2020)
	CNN		• Distinguishing tongue characteristics and diagnosing TCM syndromes • Tracking disease progression • Evaluating the effectiveness of interventions	Wang et al. (2022c)
	Transfer Learning、CNN		• Precise symptom and disease diagnosis	Wang et al. (2022b)
	SVR、RF、AdaBoost、GBRT	Tongue Diagnostic Tool	• Establishing Intelligent Analysis of Tongue Diagnosis Images in Traditional TCM	Li Z et al. (2022)
Machine learning	APINet、SVM、DT、KNN		• Evaluation of tongue image and tongue microbiome in gastric cancer (Diagnostic Applications)	Yuan et al. (2023)
	SVM、RF、NN		• Establishing innovative, non-invasive differential diagnostic methods • Characterization of disease fatigue and non-disease fatigue using tongue data and pulse data	Shi et al. (2021)
	DT、SVM、CNN、PCA、SIR、LR		• Development of a computer-aided system to diagnose the severity of sublingual varicose veins	Lu et al. (2022)

### 4.3 Precision treatment of TCM under digital intelligence technology

The core metabolites of TCM precision treatment in an Internet-based model encompass an AI system characterized by “identification and treatment,” a manufacturing big data cloud platform centred around “rationale, method, and prescription,” and an intelligent manufacturing system focused on “dosage, quality, and efficacy” for investigating the mechanisms underlying TCM prescriptions. The intelligent manufacturing system, with its emphasis on “dosage, quality, and efficacy,” serves as a pivotal element in examining the mechanisms of TCM prescriptions (Lin et al., 2022a). The framework of network medicine reveals the scientific basis. The network medicine framework elucidates the scientific rationale for predicting therapeutic potential in Chinese medications, establishes a foundation for deciphering the molecular underpinnings of natural remedies and forecasting disease treatments, and underscores the efficacy of TCM in alleviating symptoms (Gan et al., 2023; Lu et al., 2021) integrated network pharmacology, transcriptome and AI analyses to elucidate the formula Ning Lung Ping Blood Decoction’s molecular mechanism in treating acute respiratory distress syndrome. Guang et al. (2023) used a combination of data mining and network pharmacology to explore the potential mechanism of Zuo Gui Wan in the treatment of hepatocellular carcinoma (Zhu et al., 2020); used AI and network pharmacology to investigate the potential mechanism of Zuo Gui Wan in the treatment of liver cancer. The pharmacological mechanism and material basis of treating diabetes mellitus with elimination pills through AI and network pharmacology. Zhao et al. (2022b). explore the potential molecular mechanisms for the anti-hepatic fibrosis of Chinese herbal formulae to promote effective treatment for patients with hepatic fibrosis. Chen et al. (2023) have developed an early warning model based on a deep confidence network, which elucidates the biological network mechanism underlying pharmacological kidney injury induced by TCM.

TCM prescription is the link between the identification and treatment of TCM. In clinical practice, multi-herb formulas are frequently employed, emphasising the compatibility of the botanical drugs, their properties and channels of action, to treat the root cause of the disease and thereby achieve a therapeutic effect that balances yin and yang and focuses on maintaining homeostasis. In the early days, research on The compatibility of metabolite prescriptions was primarily reliant on conventional mathematical statistical tools such as orthogonal design, uniform design and dismantling research. These tools enabled the pursuit of specific research objectives, including simplifying the optimal prescription, exploring the most efficacious drug ratio and screening the effective subgroups of metabolite prescriptions. This approach not only reduced the number of experiments but also minimised the use of animals in research. However, due to the inherent limitations of the aforementioned methodologies, the application of these methods is predominantly focused on the research of small metabolite prescriptions (Zhou et al., 2010). Consequently, the compatibility rules and pharmacological mechanisms of a significant number of large metabolite prescriptions, which have been clinically proven to be efficacious, have not been adequately elucidated. Digital intelligence technology enables the transformation of ancient TCM books and clinical treatment experience into data, facilitating the establishment of an extensive TCM database. This technology also allows for research on the theoretical foundations of TCM, academic viewpoints of renowned doctors, TCM diagnosis and treatment, clinical techniques, prescription patterns, and clinical efficacy of TCM through the application of data mining, AI, and mathematical principles (Guo et al., 2019; Hu et al., 2022) used a deep neural network to implement text classification and model TCM evidence identification.

In recent years, deep learning has enabled the realization of an intelligent formula recommendation system for TCM formulas that integrates phenotypic and molecular information, promoting the transformation of the TCM research model from “based on experience and macroscopic” to “based on data and combining macroscopic and microscopic” (Zhou et al., 2021). In addition, they will establish a regulatory science system to regulate the application of AI in TCM (He et al., 2022a; Dong et al., 2022) proposed a symptom term mapping method (SSTM) based on sub-network using machine learning and AI technology. They developed a TCM prescription recommendation method, denoted as TCMPR, utilizing SSTM to provide personalized TCM prescriptions. This deep learning approach is capable of delving deeply into the intricate relationship between symptoms and TCM, facilitating the automated generation of TCM prescriptions, and advancing the research into intelligent TCM prescription development (Hou et al., 2023). Chen et al. (2023) evaluated the safety and appropriateness of the GPT-4 model in generative AI for generating TCM prescriptions, providing technical support for the rational use of clinical TCM (Li et al., 2023d). demonstrated that machine learning can be combined with TCM constitution classification in predicting insomnia severity to develop more personalized and effective interventions and improve patient outcomes (Duan, 2024). employed the ERNIE model in conjunction with the sequence-to-sequence approach within deep learning to develop a model addressing the prescription generation challenge in clinical patient consultations. This model facilitates auxiliary diagnosis and prescription recommendations for TCM, thereby enhancing the quality and efficiency of TCM clinical consultations.

The application of numerical intelligence technology in TCM clinical diagnosis, mining the complicated relationship of “disease-evidence-disease” in clinical diagnostic data to improve the standardization and objectivity of TCM diagnosis (Liu et al., 2024; Song et al., 2021). In the context of intelligent diagnosis in TCM, it is essential to understand the relationship between “disease - evidence - syndrome” in clinical diagnosis data to improve the standardization and objectivity of TCM diagnosis. Researchers have already completed prescription recommendations and personalized intelligent diagnoses guided by the concept of somatic TCM consultation (Tian et al., 2023), summarized in Table 6.

**TABLE 6:** Clinical diagnosis and intelligent dialectics in TCM.

AI algorithm	Key technology	Typology	Sketch	Bibliography
AI	SDM	TCM Clinical Diagnosis	• Designing Te charts for symptoms and evidence • Accurate differentiation of syndromes	Li M et al. (2022)
	ANN		• Development of a prediction model for dyslipidaemia classification • Translating evidence-based diagnosis in TCM into prediction and classification by AI • Development of diagnostic rules for MOPS dyslipidaemia	Liu J et al. (2023)
	SDM		• Similarity Algorithm Based on Segmentation and Weights • Improving the efficiency of smartly guided consultations	Yu and Liu (2023)
	Network Pharmacology		• The UNIQ system is a molecular network navigation system for Western and TCM. • Interpreting the Scientific Principles of TCM • Precision Diagnosis and Treatment and Precision R&D of TCM	Lin et al. (2022b)
Machine learning	NNA、NLP	TCM Clinical Diagnosis	• Promote standardization of signs/symptoms	Lin et al. (2019)
	SVM、DT		• Achieved higher performance in medical information retrieval • Automated Diagnostics for TCM EHR.	Liang et al. (2019)
	SVM、DT、NN、		• Exploring TCM Diagnosis • Promote research on the categorization of TCM patients	Zhao et al. (2015)
	NLP		• Natural language processing techniques for processing free-form electronic health record notes • Improved diagnostic accuracy and generalization	Zhang et al. (2020)

TCM prescriptions demonstrate clinical efficacy and persuasiveness; however, there remains a paucity of scientific elucidation regarding the pharmacodynamic active metabolites and their complex interactions within the human body. Additionally, there is a need for the development of systematic evaluation models to validate these therapeutic interventions further. Some scholars have adopted digital intelligence technology to identify the pharmacodynamic substances and quantitative analysis of the pharmacological effects of TCM prescriptions to better understand their mechanisms of action and scientific connotations (Qian et al., 2023). The advancement of virtual screening technology enhances the effectiveness and precision of the drug discovery process et al., 2022). The methods for predicting the pharmacological properties of TCM include virtual screening, pharmacophore modelling and machine learning (Han et al., 2018). Currently, TCM Bank is the most comprehensive, downloadable and largest non-commercial TCM database, allowing free exploration of relationships between TCMs, metabolites, gene targets and related pathways or diseases (Lv et al., 2023; Liang et al., 2022) established random forest (RF) and support vector machine (SVM) models for the prediction of anti-SARSCoV-2 activity by using machine learning methods to discover new anti-novel coronavirus metabolites and medicinal plants from TCM (Xu et al., 2021). utilized network pharmacology, enhanced by deep machine learning techniques, to elucidate the mechanism of action of the selected TCM prescription. Their findings, validated through both *in vitro* and *in vivo* experiments, identified quercetin, kaempferol, baicalein, and chuanchengpiin as the pivotal active metabolites. These results imply that the therapeutic approach of fortifying the spleen and invigorating vital energy may serve as a promising adjutant treatment for gastric cancer. Artificial neural networks and various algorithms were utilized to analyze and extract information on the medicinal properties and effectiveness of TCM as documented in the literature. This analysis also aimed to identify correlations between the medicinal properties of TCM and their efficacy, providing valuable data to predict the effectiveness of Chinese medicinal formulas. Furthermore, AI prediction models were developed to assess the similarity of TCM metabolites, enabling speculation on the identification of Chinese medicinal properties (Li et al., 2022f). Utilizing network pharmacology to construct the “active metabolite-disease-target-pathway” network, this approach facilitates the analysis of active metabolites, pathways, targets, and their interactions with diseases. This aids in elucidating the pharmacological mechanisms of drugs, uncovering the pharmacodynamic metabolites of TCM, predicting potential drug targets, and enhancing the efficiency of active metabolite screening in TCM (Hu et al., 2023). The active metabolites of TCM can be employed to enhance the efficiency of screening, as summarized in Table 7.

**TABLE 7:** Potential TCM active metabolites identified by Digital Intelligence Technology.

AI algorithm	Key technology	Research purpose	Findings	Bibliography
Machine learning	CNN	Discovery of herbal medicinal substances and verification of pharmacological effects	• Establishing the time-dose-effect relationship • Identification of the pharmacodynamic substance base, evaluation of pharmacodynamic effects	He et al. (2023a)
	SVM、RF、KNN		• Demonstrates that populin, rhubarb acid, kawakawa chenopodium and fexofenadine are highly synergistic with celecoxib	Sun et al. (2023)
	SVM、RF、ANN		• Identification of three candidates (larch resin alcohol, Tricin and 4′-desmethyl epigallocatechin) • Molecular dynamics simulations for preliminary exploration of binding properties	Yang et al. (2022a)
	Unsupervised Machine Learning 、Heterogeneous Networks	Explore the potential active metabolites of TCM and screen for effective traditional TCM.	• Establishing links between metabolite functional modules and drug-activated cellular processes	Guo et al. (2020)
	RF、SVM、Molecular Fingerprints		• A total of 1,011 active anti-novel coronavirus metabolites were predicted • Six metabolites with potent activity were demonstrated in anti-novel coronavirus experiments	Liang et al. (2022)
	RF		• Identify potential liver-protecting herbs	He et al. (2022a)
	SVM、RF、UC、WGCNA、		• Five core targets were identified (CXCL2, DUOX2, LYZ, MMP9 and AGT) • Red Astragalus (Astragalus membranaceus), Bitter Ginseng (Ginseng vulgaris), Cotyledonary Fringes (Vasicine) and Pomegranate Bark (Pomegranate Pelargonium) relieve abdominal pain and blood in stools during active UC.	Ou et al. (2023)
	RWR、Entropy Weight Method		• Predicting the active metabolites in Alzheimer’s disease (AD) and screening for effective Chinese herbs (Danshen, Bone Teng, and Chai Hu) and an effective metabolite formula, Yiqi Tongyi Tang	Wu et al. (2023)
	AB、kNN、CT、RF		• Identification of neuroprotective metabolites in Xiao Xu Ming Tang: baicalein and prim-O-glucosyl simefugin (POG)	Yang et al. (2019)
AI	AI	Development of a Chinese herbal formula for non-severe novel coronavirus infection (COVID-19) and validation of the mechanism of action	• It was demonstrated that the ethanol extract of Arctium lappa bark drink inhibited the expression of several pro-inflammatory genes	(W. Q. Huang, et al., 2024)
,Bioinformatics and virtual screening techniques	GEO、AI	Searching for non-small cell lung cancer-related genes and related anti-non-small cell lung cancer herbal medicines	• Validation of 10 key genes, including KIF20A, NUF2, TTK, TPX2, TOP2A, CDC6, DLGAP5, NCAPG, CCNB1, and KIF11, associated with non-small cell lung cancer • Exploring herbal composition to herbal medicine prediction based on improved algorithms	Zhang, (2023b)
Deep learning	ZVSegNet、HRNet	Development of medicines for conservation therapy	• Identification of cornflowerin (CyCl) as a new Keap1 inhibitor against adriamycin-induced cardiotoxicity	Liu C et al. (2023)
	DrugBAN、Molecular Docking	Improving the identification and screening efficiency of TCMs	• Prediction of affinity for dipeptidyl peptidase-IV (DPP-IV) for 11,549 metabolites in the Traditional TCM Systematic Pharmacology Database (TCMSP) • Screening of genipin 1-gentiobioside as a novel DPP-IV inhibitor	Liu H et al. (2023)

The integration of machine learning, deep learning, and other AI methodologies in TCM research is poised to become a cutting-edge research frontier. Current research primarily converges on the development of clinical prediction models in TCM, quantitative TCM models, and the refinement of specific modelling techniques (Jing et al., 2023). It primarily focuses on three aspects: (1) probabilistic model. Adopting the correlation mining method of TCM entities based on probabilistic subject models to study the intrinsic connection and interrelationship among TCM symptoms, TCM, and aetiology and to explore the correlation between TCM dispensing laws, prescribing experiences of ageing doctors and diseases (Liu et al., 2022). (2) Deep learning models, including network topology models for drug-target interactions and expert recommendation systems based on semantic integration of TCM. (3) Logic rule-based methods.), summarized in Table 8.

**TABLE 8:** Quantitative modelling of modernized TCM.

AI algorithms	Key technology	Model name	Findings	Bibliography
Deep learning	Transfer Learning、BERT	Transfer learning model	• Generation of TCM prescriptions from medical records and TCM literature resources • Handling of text resources	Liu et al. (2022)
	Character-Level Representation and Tagging、TCMNER、	Entity Recognition (NER) model	• Improving the Performance of Named Entity Recognition Models in TCM • Presentation of character sets into self-awareness modules • Create a comprehensive NER dataset containing standard content for publications and clinical records	Liu et al. (2021)
	BTSN	A Bidirectional Temporal Concatenation Network (BTSN) Model for Computing Similarity of Traditional Chinese Texts	• Solving the problem of inaccurate TCM sub-wording	Luo et al. (2021)
	CNN	S-TextBLCNN model for efficacy classification of traditional TCM prescriptions	• Analyzing the relationship between TCM efficacy and formulation efficacy • Exploring the inner workings of recipe combinations	Cheng et al. (2021)
	CGNA、MTFE、NET	A Multi-Granularity Text-Driven NER Model Based on Conditional Generative Adversarial Networks (MT-CGAN)	• Implementing a small annotated corpus • Extracting rich semantic and syntactic information from TCM texts	Ma et al. (2022)
	DL	A Rule and Model-Based Framework for Intelligent Genetic Prototyping System for Famous Elderly Doctors	• Inheriting the academic thinking and clinical experience of renowned veteran doctors • Conducting a case study of paediatric asthma treatment	Ren et al. (2023b)
	BiLSTM-CRF	A semi-supervised model for extracting TCM clinical terms using feature words	• Extraction of clinical terms such as TCM, symptoms, patterns, diseases and prescriptions	Liu et al. (2020)
	MGCN	(MGCN) model for prescription recommendation	• Improving the accuracy of TCM prescription recommendations • Provide accurate TCM prescription recommendation programme	Zhao et al. (2022a)

## 5 Discussion: the road to modernisation of TCM

### 5.1 The road to modernization of the quality of TCM

The quality of TCM is the cornerstone of its modernization. The evaluation of TCM quality encompasses the entire process from planting and processing to concocting, production, packaging, storage, and use. The current Chinese Pharmacopoeia includes regulations and standards for 2,711 types of Chinese medicinal materials, primarily focusing on identification, inspection, and qualitative and quantitative control of the metabolites in these materials. However, measuring and identifying the active metabolites of Chinese medicinal materials based on efficacy and safety, discovering new pharmacodynamic indicators in these materials, and evaluating the scientific basis of cultivation practices as well as the regional, diverse, and variable nature of TCM resource development still poses many challenges.

With the advancement of interdisciplinary fields, the quality control of TCM is gradually transcending the boundaries of traditional medicine and deeply integrating with computer science. The TCM industry is developing in the direction of quality improvement, clinical value and targeted quality and technological innovation. Digital intelligence technology can be used to scientifically guide the cultivation of TCM, improve the automation and intelligence of the production process, and ensure the safety and effectiveness of TCM production through data analysis. China’s “14th Five-Year Plan” promotes the construction of a TCM traceability system, leveraging information technology to achieve automatic identification of Chinese medicinal materials and decoctions, and building an information-based traceability system to ensure the traceability of the sources and destinations of these materials. The complexity of TCM types, metabolites, sources, and processing methods presents significant difficulties in data collection and management, which in turn affects the accuracy of AI models. High-quality, standardized data sets are the basis for training models, and the heterogeneity and lack of data may weaken the performance of the model. Complex machine learning models (especially deep learning models) often lack interpretability, and there is an urgent need to develop more transparent and easy-to-interpret algorithms. How to effectively integrate different types of data (such as chemical data, clinical data, and genetic data) to improve the overall performance of the model is still a hot topic.

Therefore, the application of artificial intelligence in TCM quality control is still in its nascent stage. To ensure the authenticity and accuracy of TCM quality, it is essential to continuously optimize model structures, improve data quality, and develop more scientific and rational basic algorithms. In the future, more attention should be paid to experimental verification and practical application scenarios. Through AI technology, it can assist in the quality assessment of medicinal materials, composition analysis and fingerprinting, pollutant detection and safety assessment, production process monitoring and optimization, and the quality and safety assessment of TCM to promote the modernization of TCM quality control. Development.

### 5.2 The road to modernization of TCM for the treatment of diseases

As a medical system with a history spanning thousands of years, TCM has demonstrated remarkable clinical efficacy and possesses unique scientific value. With the rapid advancement of machine learning and deep learning algorithms, an increasing number of text classification techniques are being applied to the dialectical research of modern TCM. AI has the potential to accurately reveal the associations between complex diseases and TCM and to identify active substances for disease treatment. The inheritance and innovation and development of TCM should first focus on the in-depth mining of massive amounts of TCM data such as ancient and modern TCM classics, literature, famous medical cases, and clinical diagnosis and treatment experience. Due to the huge density of these data and information, there are still a large number of basic mechanisms in modern TCM that are not clear, such as the synergistic mechanism of acupoints, *etc.*, which urgently need to be in-depth. Dig and interpret to promote the development of disciplines. TCM data has the characteristics of multi-source, composite, heterogeneity, *etc.*, and is unstructured, subjective, and ambiguous data. Its quality and quantity directly affect the effect of AI application. It must be structured and standardized before AI technology can be applied for further mining and analysis. Therefore, the inheritance of TCM and the inheritance of TCM are related to the development of TCM. The development must be led by the standards of smart TCM, which is also the result of the basic, strategic, and global decisions of the standards. However, several challenges remain in the application of AI to TCM for disease treatment: the imperfect standardization system of TCM, the limited scale of basic data, and the insufficient incorporation of TCM principles into algorithms and diagnostic and treatment models. In addition, the in-depth mining of massive amounts of data in TCM is a systematic project, which requires the cooperation of the government, medical institutions, scientific research institutions and AI enterprises. The government has issued policies to support and encourage open data sharing, unified management and analysis of clinical data, and medical institutions provide clinical data and resources. Scientific research institutions carry out data mining and analysis work, and all parties can work together. Realize the in-depth mining of massive amounts of data on TCM and promote the inheritance of TCM.

Currently, the TCM industry faces a shortage of composite talents who are proficient in both TCM and AI technology. The emergence of code-free AI technology has significantly lowered the technical barriers, enabling TCM researchers to build data analysis, prediction, and decision-making systems without extensive programming knowledge. Digital intelligence technology has played a crucial role in elucidating the compatibility laws of TCM metabolite composition and their underlying mechanisms, thereby promoting the objectification and standardization of metabolite research processes. However, most existing research focuses on the compatibility relationships and strengths between drugs, often neglecting the influence of factors such as dosage, usage, and preparation methods on the compatibility of metabolite prescriptions.

Although AI excels in analyzing multi-metabolite, multi-target, and multi-pathway interactions, its predictive analyses are primarily based on chemical composition or active metabolites, overlooking the complex chemical interrelationships within the human body. To gain a deeper understanding of the mechanisms of TCM action in the body, predictive results need to be integrated with techniques such as pharmacokinetics and metabolomics. As clinical treatment data accumulate, digital intelligence technology can be utilized for trait identification, the search for bioactive substances, and the formulation of standards in TCM. We need to build a multi-dimensional technology system that combines emerging technologies with multidimensional fields such as TCM, clinical and diagnostic, in-depth exploration of the deep mechanisms of TCM, and promote interdisciplinary innovation and development. Relevant staff use artificial intelligence and big data technology to develop a code-free test system, build an open, shared, and intelligent test platform, reduce the technical threshold, and improve the efficiency and accuracy of testing.

The research on the digital intelligence of dialectical treatment of TCM for the precise diagnosis and treatment of TCM, one is the need to collect effective diagnosis and treatment information that truly reflects the process of dialectical treatment, and realize standardization and digital collection to improve the accuracy and reliability of diagnostic information; the second is to digitize the “disease-evidence” multivariate data to form multivariate diagnosis and treatment data and reliable evidence waiting diagnosisThe connection mechanism is guided by the curative effect, and the digital characterization of “disease-evidence-prescription medicine” is realized to improve the level of intelligent dialectical treatment; the third is to quantitatively characterize the evolution of the symptoms of “not sick-already sick-becoming sick” according to the progress of the disease, so as to enhance the precision diagnosis and treatment capabilities of TCM. The integration of TCM and AI has expanded its applications in the fields of active substance screening, drug response prediction, drug interaction analysis, and new drug development in TCM. Establishing a methodological framework for the application and supervision of AI in TCM, and systematically clarifying the modern scientific connotations between formula compatibility and efficacy, represent important future directions. Given the inherent ambiguity, systematization, and complexity of TCM, the use of AI technology is expected to break through traditional developmental constraints and establish a new model for the inheritance, innovation, and development of TCM in the digital space.

## Abbreviations

AI, Artificial Intelligence; ANN, Artificial Neural Network; BP, Backpropagation; CNN, Convolutional Neural Network; GC, Gas Chromatography; GCN, Graphical Convolutional Neural Network; GCN, Graphical Convolutional Neural Network; KNN, K-Nearest Neighbour; LC-MS, Liquid Chromatography-Mass Spectrometry; NMIR, Near/Mid-Infrared Spectroscopy; NMR, Nuclear Magnetic Resonance; REIMS, Rapid Evaporation Ionization Mass Spectrometry; RF, Random Forest; SVM, Support Vector Machine; TCM, Traditional Chinese Medicine; UV-Vis, Ultraviolet-visible; IR, Infrared.
